# Correction to: Does this patient have Pheochromocytoma? a systematic review of clinical signs and symptoms

**DOI:** 10.1186/s40200-017-0324-4

**Published:** 2017-10-16

**Authors:** Akbar Soltani, Mandana Pourian, Babak Mostafazadeh Davani

**Affiliations:** 1 Evidence based Practice Research Center, Endocrinology and Metabolism Research Center, Tehran University of Medical Sciences, Tehran, Iran; 2 Endocrinology and Metabolism Research Center, Endocrinology and Metabolism Clinical Sciences Institute, Tehran University of Medical Sciences, Tehran, Iran

## Correction

This article [1] was unintentionally published twice in this journal, by the same authors. The following should be considered the version of record and used for citation purposes: "Soltani et al., Does this patient have Pheochromocytoma? a systematic review of clinical signs and symptoms, Journal of Diabetes & Metabolic Disorders (2016) 15:6, 10.1186/s40200-016-0226-x". The duplicate article [2] "Pourian et al., Does this patient have Pheochromocytoma? A systematic review of clinical signs and symptoms, Journal of Diabetes & Metabolic Disorders (2016) 15:11, 10.1186/s40200-016-0230-1" is to be ignored. Editor-in-Chief Prof Bagher Larijani apologizes to the readers of the journal for not detecting the duplication during the publication process.”
